# Response to a letter to the editor titled, “Weight loss in patients with obstructive sleep apnea: an interventional procedure”

**DOI:** 10.1007/s11325-020-02154-6

**Published:** 2020-10-28

**Authors:** Matsusato Tsuyumu, Tadao Tsurumoto, Jiro Iimura, Tsuneya Nakajima, Hiromi Kojima

**Affiliations:** 1 Department of Otorhinolaryngology, Tokyo Dental College Ichikawa General Hospital, Chiba, Japan; 2 Department of Otorhinolaryngology, Jikei University School of Medicine, Tokyo, Japan; 3 Department of Otorhinolaryngology, Jikei University Daisan Hospital, Tokyo, Japan

To the Editors-in-Chief:

We thank Dr. Tomoyuki Kawada for his attention to our research article “Ten-year adherence to continuous positive airway pressure treatment in patients with moderate-to-severe obstructive sleep apnea” [1]. Dr. Kawada’s letter questioned treatment limited to continuous positive airway pressure (CPAP), and stated that weight control for patients with OSA is important for the treatment. However, his letter also admitted that long-term weight control therapy is difficult and recommends a combination of weight control and CPAP.

As Dr. Kawada’s letter suggests, OSA treatment with CPAP is not a fundamental method for improving OSA, and many previous studies have demonstrated that weight loss can reduce the severity of OSA in patients. On the other hand, Iftikhar et al. conducted network meta-analysis of 80 randomized controlled trials (RCTs) and compared treatments with CPAP, a mandibular advancement device, exercise, and weight loss. They stated that CPAP was the most effective in reducing the apnea-hyperpnea index (AHI), and the patients would also benefit from exercise [2]. Another perspective is a previous study showing that a combination of CPAP and weight loss can reduce cardiovascular risk [3].

Many patients with OSA are overweight or obese, and the effectiveness of CPAP and weight control, respectively, on OSA has been confirmed in many previous studies. The treatment goals for each patient with OSA will determine the best remedies, but we agree that CPAP and weight control would be beneficial combined therapies to improve pathophysiology of OSA, improve AHI, and reduce cardiovascular risk.

While overweight or obesity can be a causal factor for OSA, this sleep condition is also known to occur in patients with low to moderate BMI. In other studies, these patients were treated with methods such as CPAP or lateral sleep [4, 5]. Our study, which included 56 patients (30.9%) with a BMI of less than 25 kg/m^2^, showed that a high body mass index (BMI) in patients with OSA was an independent positive predictor of long-term use of CPAP. This result also suggests that some patients with OSA and low BMI might be unable to use CPAP for the long term. Further research is needed to explore treatments for OSA in patients with low to moderate BMI.
